# The Global HIV/AIDS Vaccine Enterprise: Scientific Strategic Plan

**DOI:** 10.1371/journal.pmed.0020025

**Published:** 2005-01-18

**Authors:** Coordinating Committee of the Global HIV/AIDS Vaccine Enterprise

## Abstract

The development of an HIV vaccine remains one of the most difficult challenges confronting biomedical research today. A new international collaboration shares its plan to address the challenge

## Introduction

In June 2003, an international group of scientists proposed the creation of a Global HIV Vaccine Enterprise [1]. The authors invited discussion of this proposal, and challenged scientists to identify new strategies and mechanisms to accelerate the global effort to develop a safe and effective HIV vaccine. This paper describes the processes that led to agreement on the major roadblocks in HIV vaccine development, summarizes current scientific priorities, and describes an initial strategic approach to address those priorities. Specific research is not prescribed. Rather, the intent is to stimulate both researchers and funders to explore new, more collaborative, cooperative, and transparent approaches to address the major obstacles in HIV vaccine development identified in the plan, in addition to continuing the productive, high-quality programs already underway.

The major difficulties encountered in the development of an HIV vaccine are scientific, not organizational.

The motivation behind the proposal for a Global HIV/AIDS Vaccine Enterprise was the recognition that development of an HIV vaccine remains one of the most difficult challenges confronting biomedical research today [2,3]. Fortunately, scientific progress has created new opportunities that could be harnessed more effectively through global coordination and collaboration. These new opportunities include an expanded HIV vaccine candidate pipeline, improvements in animal models, a growing database from clinical trials, and the availability of new quantitative laboratory tools that make comparisons among vaccine studies feasible. Confronting major roadblocks and harnessing these new opportunities requires an effort of a magnitude, intensity, and design without precedent in biomedical research, with the Human Genome Project as a potentially useful model [4]. More specifically, the critical scientific insights generated by the creativity of individual investigators, as well as small groups and individual networks, could be significantly augmented by a properly organized, managed, and systematized international effort targeted on the design and clinical evaluation of novel HIV immunogens. An international collaborative effort that addresses a shared scientific plan, provides information exchange among groups, links clinical trials with standardized laboratory assays and evaluation in animal models, applies new knowledge to improvements in vaccine design in an iterative manner, and supports a transparent process for decision making in all aspects of vaccine discovery, design, development, and clinical testing will prove critical to success.

The Global HIV/AIDS Vaccine Enterprise represents a novel paradigm to seek and identify international agreement on the critical roadblocks for developing an HIV vaccine and on creating a shared scientific plan that addresses those roadblocks (see Box 1). The Enterprise proposes to coordinate efforts at a global level, facilitate use of common tools and technologies, and help ensure access to optimized resources. Furthermore, the Enterprise approach is a way of behaving as a global community of problem-solvers, more openly sharing information, ensuring that the shared scientific plan is implemented, and basing decisions on evidence rather than advocacy.

**Box 1. Key Points in the Scientific Strategic Plan**

**Figure 1:**
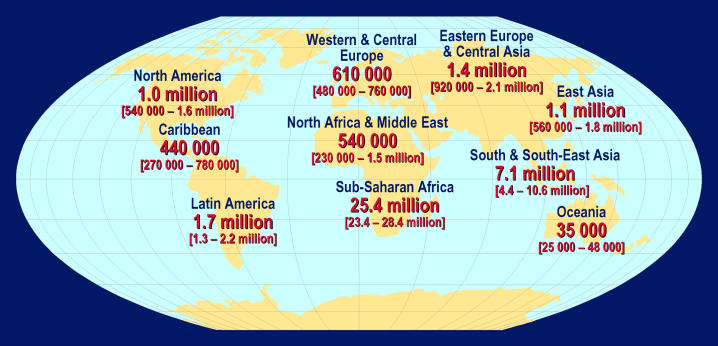
Adults and Children Estimated to Be Living With HIV as of the End of 2004 (Total: 39.4 [35.9–44.3] million) (Map: UNAIDS/WHO)

**Figure 2:**
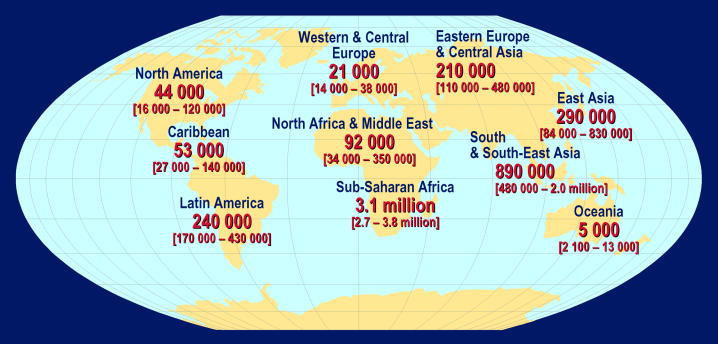
Estimated Number of Adults and Children Newly Infected with HIV during 2004 (Total: 4.9 [4.3–6.4] million) (Map: UNAIDS/WHO)

**Figure 3:**
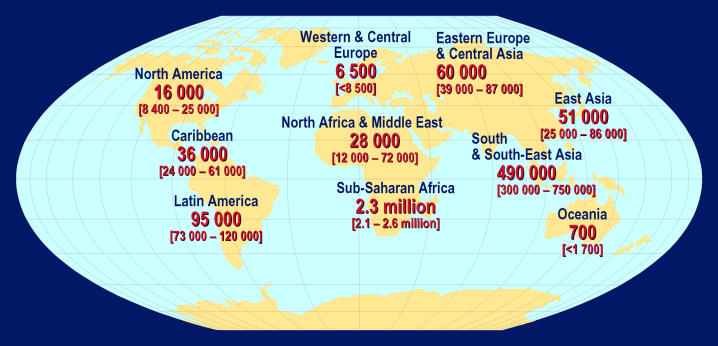
Estimated Adult and Child Deaths from AIDS during 2004 (Total: 3.1 [2.8–3.5] million) (Map: UNAIDS/WHO)

• More new HIV infections and AIDS deaths occurred in 2004 than in any prior year (Figures 1–3). A vaccine is critical for the control of the pandemic.

• Development of an HIV vaccine is one of the world's most difficult and important biomedical challenges.

• Harnessing new scientific opportunities for HIV vaccine development will require an effort of a magnitude, intensity, and design without precedent in biomedical research.

• The Global HIV Vaccine Enterprise is an alliance of independent organizations committed to accelerating the development of a preventive HIV/AIDS vaccine based on a shared scientific plan.

• The scientific strategic plan was developed with the collaboration of over 140 scientists and other participants from 17 countries and several international organizations.

• The plan identifies critical unanswered scientific questions along the critical path for vaccine discovery, from antigen design to the conduct of clinical trials.

• Novel vaccine candidates need to be designed to induce high levels of broadly reactive and persistent immune responses against HIV strains circulating in different parts of the world.

• Standardization and validation of high-throughput laboratory assays conducted under GLP will allow comparison of results from different vaccines, which is a linchpin of rational decision making in vaccine development.

• The Enterprise will encourage decision makers to establish clear and transparent processes to identify and prioritize the most promising vaccine candidates.

• The Enterprise will seek to engage the best researchers who are willing to work in a highly collaborative manner and to dedicate the majority of their efforts to solve the fundamental roadblocks in HIV vaccine development.

• To mount an accelerated global search for a safe and effective HIV/AIDS vaccine, annual funding for such research should double—to US$1.2 billion per year.

• Several founding partners of the Enterprise have already committed, or are planning to commit, new funding to support the proposed Enterprise activities, and to create a culture of mutual accountability for the effective implementation of the scientific strategic plan.

• Enterprise activities are guided by an international Coordinating Committee, supported by different technical expert groups, including representatives from funders and implementers of HIV vaccine R&D.

It must be emphasized, however, that the major difficulties encountered in the development of an HIV vaccine are scientific, not organizational, and arise directly from the complexities of HIV and AIDS. “Small science” should not be replaced with “big science.” Both approaches must be undertaken. Creation of research environments that support the creativity both of individual investigators and of larger, collaborative efforts will accelerate the scientific breakthroughs needed to successfully develop a safe and effective HIV vaccine.

## Scientific Priorities

### Prioritization process

In August 2003, the authors of the Enterprise proposal invited a group of leading scientists, public health experts, and policy makers to meet at the Airlie House in Warrenton, Virginia, United States, to refine the vision for the Enterprise. The Airlie group agreed that the Global HIV/AIDS Vaccine Enterprise should be developed as an alliance of independent organizations committed to accelerating the development of a preventive vaccine for HIV/AIDS through implementation of a shared scientific strategic plan, mobilization of additional resources, and greater collaboration among HIV vaccine researchers worldwide [5].

The subsequent initial planning phase of the Enterprise involved leading government research agencies, private industry, non-governmental organizations, and funders involved in HIV vaccine research and development (R&D) activities, including the Bill & Melinda Gates Foundation (BMGF), the International AIDS Vaccine Initiative (IAVI), the National Agency for Research on AIDS of France (ANRS), the United States National Institutes of Health (NIH), the United Nations Joint Programme on HIV/AIDS (UNAIDS), the World Health Organization (WHO), and the Wellcome Trust. The Enterprise is expected to grow with time and include additional organizations and research groups willing to contribute to the implementation of its scientific strategic plan. A Steering Committee composed of representatives from several of the founding organizations provided guidance and coordination, with the BMGF serving as interim Secretariat.

Six Working Groups involving more than 120 participants from 15 countries, the WHO, and UNAIDS were formed to develop the scientific plan of the Enterprise. These Working Groups met from January to April 2004, identified critical unanswered questions, and proposed actions to address them. In May 2004, the Steering Committee of the Enterprise analyzed the recommendations from the Working Groups and identified the scientific priorities for initial action.

Several common themes emerged from the Working Groups. There was clear agreement on the key scientific challenges, as well as strong consensus that the HIV vaccine field has progressed to a point where it should be possible to answer some of the persistent questions more definitively. To meet these challenges, the Working Groups called for enhanced access to reagents and technologies, adequate resources, and strengthened human capacity in several key areas, especially in developing countries, where clinical trials need to be conducted. There was also agreement that the present way of doing business, which centers primarily on individually led research groups or networks, needs to be supplemented by establishing focused, collaborative structures and providing access to common standards and technologies, which would enable comparison of data and candidate vaccines. This would, in turn, support a rational process for decision making to advance candidate vaccines through the different phases of evaluation.

### Vaccine discovery

One immediate goal is to design HIV candidate vaccines that consistently induce potent, broadly reactive, persistent neutralizing antibodies, as well as memory T cells that suppress viral replication and prevent escape of virus from immune control [6,7]. Additional research is also needed to identify how mucosal [8] and innate [9,10] immunity could be harnessed to develop effective HIV vaccines. The ability to develop effective vaccines would be greatly enhanced by an understanding of what specific immune response or responses correlate with vaccine-induced protection [11].

The current state of the art suggests a two-pronged strategy to accelerate the development of a safe and effective HIV vaccine. One component should center on candidate vaccines already in the pipeline, nearly all of which are designed primarily to induce T cell responses. In some animal models these T-cell-inducing candidate vaccines suppress post-infection viremia and prevent or delay HIV disease, rather than prevent infection [12,13]. In studies of individuals infected with HIV, viral load correlates with efficiency of transmission [14], suggesting that a vaccine capable of suppressing viral load might reduce HIV transmission.

The second component should address critical gaps in scientific knowledge through carefully designed, focused, coordinated, and well-supported approaches. The fruits of this work will be a clearer understanding of what properties are needed for a successful vaccine and how to design candidates that incorporate those properties.

Scientific areas in which a more collaborative and organized Enterprise approach will be beneficial include the following: vaccine design based on the characteristics of recently transmitted viruses, evaluation of immune correlates of protection in animal models, and design of novel candidates vaccines that induce neutralizing antibodies and T cell immune responses.

Identifying which T cell candidate vaccine is most promising has become an urgent priority.

#### Vaccine design

Strategically, vaccines that are designed based on recently transmitted viruses hold the best hope of inducing relevant immune responses against currently circulating strains. Recent data suggest that the subset of viral strains that are sexually transmitted has unique genetic and antigenic properties, including greater susceptibility to neutralization than the bulk of circulating virus [15]. While such observations require confirmation, newly transmitted viruses are nonetheless the crucial targets of vaccine-induced immunity. Therefore, virological and immunological characterization of acute/early HIV infection should inform the design of vaccines and also guide the design of trials capable of determining whether immunization impacts virus levels and the course of HIV infection.

To address these issues, a representative number of virus strains derived from recently infected individuals representing those populations who will participate in vaccine efficacy trials, including populations in developing countries, should be obtained. These virus isolates should be subjected to a comprehensive genetic and biologic characterization, together with an analysis of host immune responses and the genetic background of those populations participating in the clinical trials.

This continuous and ongoing effort will require a multidisciplinary global approach, linking investigators who are conducting epidemiological and cohort studies (to allow for detection of acute/early infections), laboratory scientists working on the virology and immunology of acute/early infection and on the genetic characterization of affected human populations, vaccine designers and manufacturers, and clinical trialists. In addition, systems for data management and analysis that will facilitate the rapid translation of new information into improved vaccine designs need to be developed.

#### Immune correlates

Nonhuman primate models of AIDS offer opportunities to evaluate potential correlates of immune protection. While a particular immunization strategy that works in animal models may or may not predict protection in humans, important insights into potential immunologic mediators of protection would result from such studies. Several experimental vaccines induce varying degrees of protection against simian immunodeficiency virus (SIV) or chimeric simian/human immunodeficiency virus in rhesus macaques. In particular, studies using models in which a very high level of protection from acquisition of infection was achieved are needed, i.e., immunization with live attenuated SIV and attenuation of SIV infection by short-term antiretroviral treatment administered immediately after SIV inoculation [16,17].

To facilitate this process, assays for many different immune responses to SIV and chimeric simian/human immunodeficiency virus need to be standardized, validated, and made available to different research groups. Likewise, agreements need to be reached on those monkey challenge models that most closely resemble HIV transmission and infection in humans. Large numbers of animals will be needed to achieve statistical significance for experimental findings [18], which in turn will require expanded primate breeding and housing capability. A multidisciplinary approach that links virologists, immunologists, vaccine developers, primatologists, data and project managers, and others will be needed.

#### Neutralizing antibodies

There is increasing agreement that a successful vaccine needs to induce both humoral and cell-mediated immunity. Development of immunogens capable of inducing antibodies that neutralize primary HIV isolates from all genetic subtypes and regions of the world remains the most difficult challenge in the field of HIV vaccinology [19,20]. Success will likely require a deeper understanding of the structural motifs of the HIV envelope protein that interact with cellular receptors and/or that are recognized by broadly neutralizing antibodies [19]. This strategy will require numerous well-characterized, broadly neutralizing monoclonal antibodies, the application of peptide and carbohydrate chemistry, structural biology, and genetic engineering approaches to immunogen design, and the use of iterative approaches guided by the immunogenicity of new designs.

Given the importance of these endeavors and the uncertainty as to what path will lead to success, multiple intersecting approaches need to be explored, including, for example, the design, production, and evaluation of (1) envelope proteins that stably reveal neutralization epitopes that may be only transiently exposed during viral entry into target cells, (2) immunogens that contain rigid, stable epitopes that mimic the portion or portions of envelope recognized by broadly neutralizing monoclonal antibodies, (3) modified envelope proteins that better expose existing relevant epitopes, and (4) molecules that resemble a stabilized version of the mature envelope trimer on the virion surface. These are examples of current approaches being explored, some or all of which may prove ineffective. Additional novel ideas need to be proposed and explored.

To achieve the above objectives, new tools and technologies such as those able to detect rare, broadly neutralizing monoclonal antibodies through large-scale screening of human sera will have to be developed. In addition, the very limited existing capacity to translate structural information into stable immunogen products needs to be expanded.

#### T cell vaccines

Nearly all current vaccine candidates in the clinical pipeline are T-cell-inducing vaccines, e.g., poxvirus recombinant vectors, adenoviral vectors, DNA constructs with or without adjuvants, and lipopeptides. The ongoing effort to evaluate these products and to develop new ones is considerable [21]. Identifying which T cell candidate vaccine or vaccines are most promising has become an urgent priority. However, these evaluations are being conducted within separate preclinical research groups and, to a lesser extent, separate clinical trial networks, with the result that candidate vaccines may not be optimally compared preclinically or clinically. This approach may result in delays in identifying the most promising candidates, and it risks devoting time and resources to inferior products, although it is recognized that the specific immune responses needed for a successful vaccine remain unknown.

The identification and optimization of promising candidates will require (1) defining clear, transparent processes for decision making, (2) establishing agreement on vaccine characteristics upon which decisions should be based, (3) developing and using validated assays to assess those parameters, to allow for preclinical and clinical comparison among candidates, and (4) establishing closer coordination and data-sharing among product developers, which will accelerate the availability of critical information needed to identify and further develop the most promising candidates.

Development of an HIV vaccine remains one of the most difficult challenges confronting biomedical research today.

Research is also needed to develop improved novel T-cell-inducing candidate vaccines, especially those that avoid or otherwise circumvent anti-vector immune responses [22], and those that induce persisting high levels of immunity, especially mucosal immunity. In addition, a thorough, systematic exploration of adjuvants that markedly enhance the quantity, quality, and durability of immune responses to HIV vaccines is needed.

### Laboratory standardization

Comparison of results from preclinical and clinical studies is the linchpin of rational decision making regarding further development of vaccine candidates. Therefore, the initiation of approaches that will permit valid comparisons is crucial.

Progress to standardize and validate a limited number of T cell assays has been made within the laboratories of vaccine developers and within some partnering research networks. This approach now needs to be more broadly applied and extended to the analysis of neutralizing antibody responses. A robust infrastructure that develops, expands, and ensures broad access to quality assay technologies will allow valid comparison of data across trials and networks worldwide.

In order to achieve this goal, the following are required: (1) a decision-making process to select a set of robust assays, standardized and validated across laboratories, for measuring vaccine-induced immune responses in humans and animals; (2) wide availability of common reagents (such as peptides, control sera, and virus panels); (3) capacity for developing novel assays and reagents of potential value and for their translation to preclinical and clinical settings; (4) “core” laboratories that run selected assays and serve as a reference laboratory for satellite laboratories (clinical and preclinical work would take place in separate facilities, and clinical studies would require Good Laboratory Practices [GLP] conditions); (5) satellite laboratories located at or very near clinical trial sites to carry out a range of activities such as processing blood, storing and shipping specimens, and conducting basic immunological evaluation, and to participate in other Enterprise-organized activities such as acute/early infection studies; (6) an ongoing global quality assurance function encompassing all participating core and satellite laboratories and covering both routine safety as well as immunologic and virologic assessments; and (7) transfer of research assays and, when and where feasible, validated endpoint assays to satellite labs, including the necessary training activities.

In addition, new assay development has failed to keep pace with current understanding of the biology of the immune system and recent advances in technology. A more active program of applied research and assay development is needed to explore new concepts that would advance technical abilities and provide a better understanding of the immune responses generated by HIV vaccines.

#### Cellular immunity

Two assays are currently used for the primary evaluation and enumeration of antigen-specific T cells: Interferon-γ ELISPOT and multiparameter flow cytometry. The ELISPOT assay was initially developed to measure CD8+ T cell responses. Several observations in both mice and humans have indicated that protective immune responses will likely require stimulation of both CD4+ and CD8+ T cell effector and memory functions; it is unlikely that induction of Interferon-γ-secreting T cells alone correlates with protective immunity [11]. Therefore, additional laboratory assays measuring multiple HIV-specific cell types as well as functional capabilities will be needed to thoroughly evaluate vaccine-induced immune responses. These assays should also permit rapid assessment of the magnitude and breadth of immune responses, and enumerate the specific epitopes that are recognized.

#### Humoral immunity

Different laboratories use different assays to measure antibodies that neutralize HIV and related viruses, SIV and chimeric simian/human immunodeficiency virus. These assays vary technically, but the most widely accepted assays measure reduction in virus infectivity in cells that express the receptors necessary for virus entry. Assays that offer the greatest value are those that are validated, amenable to high throughput, low in cost, readily transferable, and that can be performed according to GLP guidelines.

The ability to measure the magnitude and breadth of neutralization against diverse HIV strains is essential to evaluating responses generated by candidate HIV vaccines. Only with multiple strains of virus can neutralization breadth be ascertained in a meaningful way. Standard panels of HIV strains are in early stages of development. Expansion or extension of current standardization and validation activities, production and provision of necessary reagents, and access to quality assurance programs are needed to ensure worldwide comparability of assay results [23]. The strains of virus incorporated into a worldwide panel need to be carefully selected to reflect the current epidemic and should include early isolates from individuals at potential vaccine trial sites [24]. Molecular epidemiological studies and elucidation of the role of genetic factors and immune responses of the host in the transmission of HIV at the population level will also help guide vaccine design and evaluation [25,26]. Another specific priority is an assessment of the neutralizing antibody response generated in the recently completed Phase III trials of HIV envelope glycoprotein 120 candidate vaccines using a global virus panel. The results would establish a baseline level of neutralization potency and breadth that is non-protective, which would be extremely valuable in reaching informed decisions about advancing future antibody-based candidate vaccines.

As more HIV candidate vaccines enter the pipeline, current capacity will be rapidly exhausted.

A major obstacle to designing a suitable global virus panel is the paucity of information on neutralization serotypes. There is general agreement that if a reasonably small number of neutralization serotypes exist, their identification would guide the creation of an optimal panel of isolates for neutralizing antibody assays and the design of polyvalent immunogens. Although there is some controversy as to whether HIV-1 neutralization serotypes exist, the magnitude of benefit that would result if serotypes were identified warrants establishment of a neutralization serotype discovery program that employs the latest technologies.

### Product development and manufacturing

Manufacture of vaccine candidates for large clinical trials and to meet eventual worldwide demand requires the development of processes for producing consistent, active vaccine batches on a large scale. Development of these bioprocesses must be integrated with analytical work (e.g., toxicity and stability testing), incorporate validated assays, and be applicable to the manufacture of sufficient vaccine to meet global needs after licensure. These processes are typically individually developed as a candidate vaccine advances from early clinical testing to late-stage evaluation and licensure. Worldwide expertise and capacity for this bioprocess development work is already limiting and exists almost exclusively in the private sector. As more HIV candidate vaccines enter the pipeline, current capacity will be rapidly exhausted.

The initial priority is to identify or establish one or more dedicated HIV vaccine bioprocess and analytical development groups that bring together the skill set and capacity to manufacture different promising candidates for clinical trials. The bioprocess development groups would also help train people and transfer manufacturing skills in whole or in part to manufacturing sites around the world. This training program would address the acute shortage of bioprocess experts.

At a later stage, building, acquiring, or contracting facilities to carry out bioprocess and analytical work and to produce several different types of candidate vaccines should be considered. Such facilities would further assist in transferring manufacturing technology to other production facilities, preferably in one or more developing countries. Decisions about which candidates a facility undertakes would be made through a well-defined, comprehensive evaluation process. The facilities could eventually be expanded to provide production capacity to launch a vaccine for public health use, should no manufacturer be available to produce the vaccine quickly upon licensure.

### Clinical trials capacity

As a growing number of HIV candidate vaccines begin to move through the clinical trials pipeline, the gap between existing global capacity and future requirements for conducting large efficacy trials has grown in magnitude and urgency, especially in developing countries. This gap in developing countries must be addressed through (1) increasing the quantity and quality of research staff, (2) establishing sustainable research facilities to support trials, and (3) expanding access to large, well-defined populations of uninfected people at high risk of HIV infection.

The acute shortage of qualified personnel is a major bottleneck to the conduct of clinical trials in developing countries.

The recommended solutions take a long-term view and are aimed at building site capacity rather than preparing for specific trials. Sites should not be confined to conducting HIV vaccine trials but should be positioned to contribute to other research of public health importance to the community and the country, including, for example, other areas of HIV research (e.g., microbicides and treatment) and/or other diseases. Additional field trial sites must be developed to be able to conduct planned and anticipated efficacy trials. Sites should be selected in a strategic, data-driven manner, and should demonstrate the ability to recruit and retain large numbers of HIV-negative volunteers from populations with substantial HIV incidence. New efficacy trial sites should be developed in regions with emerging epidemics rather than only in areas with already-established disease. “Early-warning systems” must be available to identify these newly emerging sub-epidemics. Defining optimal methods for collection of HIV incidence data from populations at potential efficacy trial sites is essential. Whenever possible, efficacy trial sites should be linked to (1) academic medical centers to enhance research capacity and help train clinical researchers, (2) accredited local and regional laboratory facilities to provide infection endpoint and safety assessments, and (3) centers that can provide appropriate care and treatment to trial participants.

The acute shortage of qualified personnel is a major bottleneck to the conduct of clinical trials in developing countries with severe or rapidly emerging HIV epidemics. Development of intellectual capacity at these sites should emphasize (1) expanding research training opportunities for personnel in the broad range of topics required to conduct high-quality clinical research, (2) establishing and adequately supporting long-term career paths for such individuals, and (3) fostering political and social environments locally and nationally that support the conduct of clinical research. Building HIV scientific and operational expertise at clinical trial sites should be linked to other HIV/AIDS research activities (e.g., identifying and characterizing incident/early HIV infections, collecting newly transmitted strains, and measuring incidence in high-risk populations).

Site development must include strategies to develop or enhance existing capacity to deliver health care, including HIV prevention, care, and treatment, to the local community participating in clinical trials. Provision of, or referral to, basic clinical services such as voluntary counseling and testing and diagnosis and treatment of sexually transmitted infections will be essential.

In addition, site development should include building skills that are ancillary but critical to the actual conduct of clinical trials, such as educating communities, building community partnerships, managing site finances, and piloting applications through regulatory decision-making processes.

### Regulatory considerations

The Enterprise must address a number of problems that currently impact the review of HIV vaccine trial protocols and that could delay future decisions regarding product licensure in developing countries. Most regulatory challenges arise from the fact that regulatory approvals are granted at the national level, but many developing countries lack the expertise, well-defined processes, clear delineation of authority, and/or other system components needed to make regulatory decisions expeditiously. As a result, new products are often licensed in these regions based on prior approval in the US or Europe and/or endorsement by the WHO. Under these circumstances, data specific to developing country populations (e.g., disease burden or childhood vaccination schedules) often do not enter into the decision making. The absence of defined pathways to approve products targeting a country's needs when a product is not also submitted to regulators in the US or Europe remains another obstacle. The Enterprise process has identified these action-item priorities: (1) harmonize and exchange information needed by regulatory bodies within the differing legal frameworks of different countries, (2) facilitate regulatory decision making, possibly using regional approaches for conducting reviews and making recommendations, (3) build regulatory capacity, (4) perform risk/benefit evaluations in the context of differing epidemic dynamics and country needs and resources, (5) identify and remove potential scientific impediments to rapid regulatory decision making, and (6) address ethical issues that interface with regulatory decision making, such as ensuring informed consent and defining the degree to which trial participants should receive a standard of care that is higher than others in their community.

### Intellectual property issues

Given the Enterprise focus on stronger collaboration, data sharing, and use of common materials and reagents, an intellectual property (IP) framework that facilitates this “enabling environment” is crucial for success. While IP issues may arise throughout the vaccine development process, at present the top priority is to stimulate early stage research and vaccine design by increasing scientific freedom to operate and sharing of data and biological materials.

Specific areas for further consideration include: (1) minimizing restrictions on freedom of operation, perhaps by early stage covenants not to litigate and followed by later stage agreements based on true valuations of IP; (2) sharing of information (including clinical trial data), materials, expertise, trade secrets, and platform technologies in a protected and secure manner while also remaining in compliance with national laws devised to prevent monopolies and insider trading; (3) recognizing the contribution of different countries to HIV vaccine development through approaches that assure affordable access to successful vaccines; and (4) maximizing access to essential technologies and inventions.

## Scientific Plan

### Scientific activities

On October 21, 2004, a group of participants from 16 countries, the European Commission, UNAIDS, and the WHO met to finalize the scientific plan and to discuss how to formulate specific actions.

Participants noted that the structure of an activity should depend on several factors, including, for example, the degree to which the activity can be predefined, the degree to which the creativity of academic researchers needs to be harnessed, and the mechanisms available to the funding organization.

A number of options were discussed, with consensus as to those that would fit various scientific priorities.

**Figure 4:**
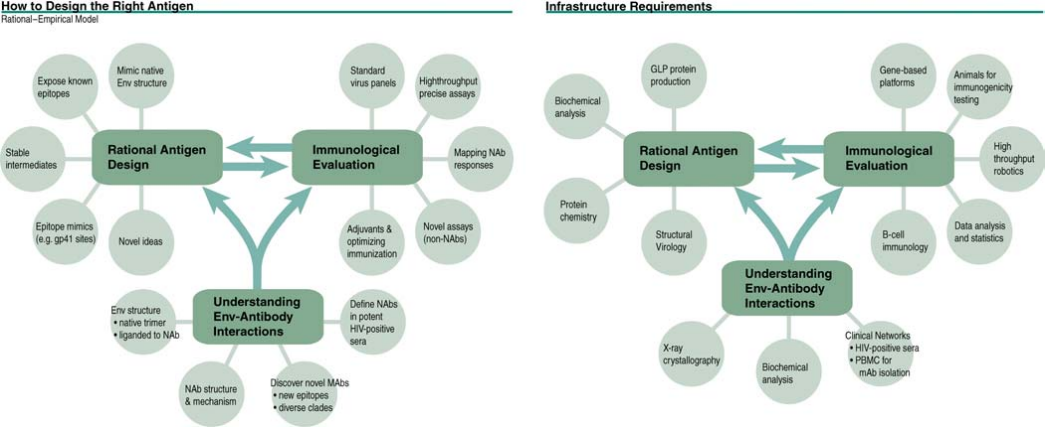
A Possible Model to Address Key Scientific Questions through an Appropriate Organizational Infrastructure (Courtesy of John Mascola; illustration: Giovanni Maki)

First, networks of focused consortia and real or virtual centers are well suited to systematically address many of the major scientific roadblocks identified in this plan. These consortia or centers would link to each other to ensure a comprehensive, systematic approach, sharing information so that each can be as productive as possible, and also to share reagents and procedures so that data among groups can be compared and, where possible, merged for analysis (Figure 4). The specific scientific areas that could be supported by consortia or centers include (1) addressing fundamental scientific problems, such as the definition of correlates of immune protection in selected animal models and the characterization of acute/early infection in potential vaccine trial sites; (2) designing and evaluating novel vaccines, such as immunogens that neutralize primary isolates, and improved T cell vaccines that avoid immunological escape and/or that induce persisting mucosal or persisting systemic responses; and (3) providing for a systematic evaluation of potential adjuvants. The success of consortia or virtual centers will depend on engaging the best researchers, getting them to work collaboratively and dedicate the majority of their effort to HIV vaccine research, resolving IP issues, obtaining support for researchers from their institutions, and keeping the group focused on specific, well-defined questions. More than one consortium may be needed for systematic coverage of vaccine design research (e.g., monoclonal-antibody-identified epitopes, native envelope, and modified envelope).

**Figure 5:**
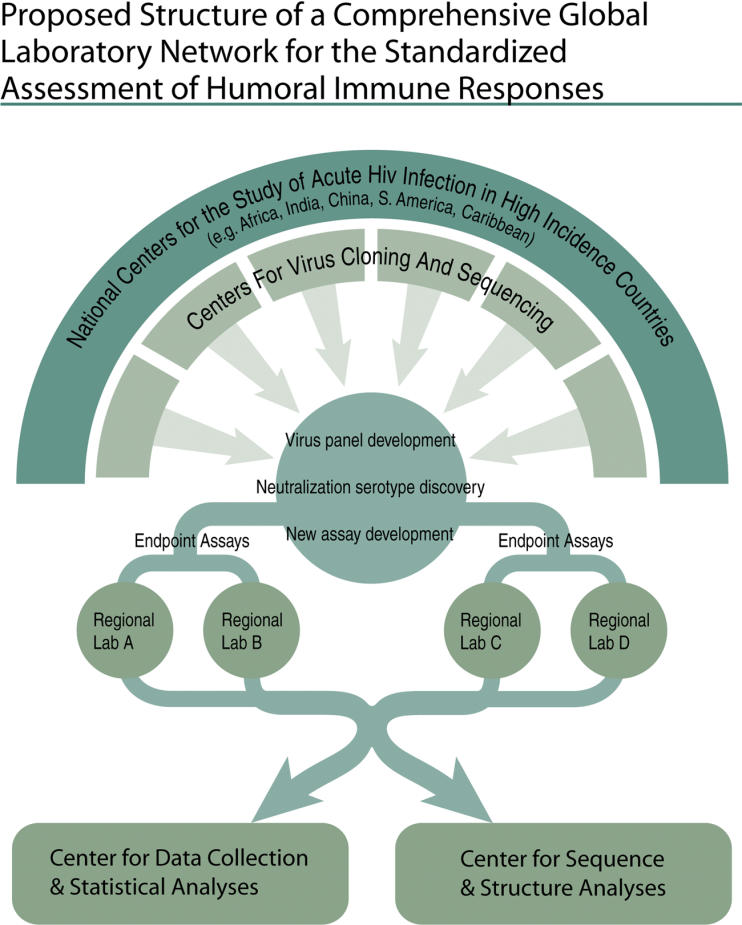
A Possible Model for a Comprehensive Global Laboratory Network for the Standardized Assessment of Humoral Immune Responses (Courtesy of David Montefiori; illustration: Giovanni Maki)

Second, a global system of central laboratories linked to satellite laboratories that work together (using GLP) would provide a range of standardized functions, help ensure the quality of clinical research, and enable comparison of data from different trials (Figure 5). Together this system could (1) conduct preclinical or clinical assays, particularly critical endpoint assays that require standardization and/or validation; (2) develop, optimize, and validate new assays and platforms; (3) transfer assays from central labs to satellite labs; (4) develop and implement a global quality control/quality assurance program and proficiency testing for assays performed at central and satellite laboratories; (5) implement vaccine-related research that requires validated assays and close cooperation and collaboration among labs globally, such as a Virus Neutralization Serotype Discovery Program, and the characterization of recently transmitted HIV isolates; and (6) contribute to the development of technological infrastructure in developing countries.

Third, a number of contract laboratories capable of developing, acquiring, storing, and distributing common reagents will prove critical to the success of collaborative research and development projects, and to ensuring reagent quality. These reagents could include (1) peptides, antisera/antibodies, and viral isolates for immune assays, including a standard panel of virus strains and sera representative of the global genetic and immunologic variability of HIV, and (2) additional broadly neutralizing monoclonal antibodies, especially from non-clade B viruses, to facilitate elucidation of the motif or motifs they recognize. These contract laboratories would be expected to work very closely with and enable the work of Enterprise consortia, centers, immune assessment laboratories, and clinical sites.

Fourth, a network of Clinical Research Training Centers in developing countries could work collaboratively to ensure development of quality trial sites. These centers would (1) conduct or facilitate training of trial site personnel in activities that are generic to the conduct of clinical trials, as well as those specific for HIV vaccine trials, for example, an HIV vaccine fellowship program for developing country scientists; (2) coordinate and work together with other Enterprise consortia or centers, such as those established to characterize acute/early infection in developing country settings or to prepare a standard panel of HIV strains representative of currently circulating viruses; and (3) share standard operating procedures, vaccine development plans, and strategies for engaging and ensuring community and political support.

Fifth, a network of individuals and companies with manufacturing experience, particularly process development expertise, could link to consortia, centers, and others involved in vaccine development to provide development and manufacturing expertise to facilitate the advancement of improved HIV vaccine candidates.

The above structures are proposed to address the initial Enterprise scientific priorities. Additional consultative groups, reference and centralized facilities, and other mechanisms may be needed to facilitate collaborative work and strengthen the global capacity for the conduct of HIV vaccine research and development as the field progresses.

Different implementing and funding agencies will need to work in close collaboration to ensure harmonious implementation of the scientific plan. Initial actions should focus on the areas of vaccine discovery and standardization of laboratory assays, which are considered critical for the success of the Enterprise and the eventual development of a safe and effective HIV vaccine. Activities to address recommendations in the areas of product development and manufacturing, clinical trials capacity, regulatory considerations, and IP issues should be launched after these initial components of the plan are under way.

Regardless of timing, each scientific endeavor needs to outline specific strategies to ensure information exchange and capacity building among the collaborating partners and institutions. The funding mechanisms employed (i.e., contracts, grants, interagency agreements, etc.) will depend on the task to be accomplished and the needs and capabilities of each funding organization. In the spirit of coordination, collaboration, and transparency promoted by the Enterprise, two or more partners may jointly support one or more activities, taking care to avoid duplication in the use of their respective resources. When a research area is jointly funded, all communication regarding goals, research plans, progress, obstacles, etc., should be openly and transparently shared among all stakeholders—funders, project managers, and researchers.

### Guiding principles

As an alliance of independent entities, the Global HIV/AIDS Vaccine Enterprise will be challenged to carry out three essential functions. One is to continue regular scientific assessments. The scientific priorities outlined in this paper will need to be monitored, re-evaluated, and updated. An evolving scientific plan must reflect lessons learned, new opportunities, and the influence of new scientific findings and new technologies. Revised versions of the scientific plan must be made fully and publicly available. The second essential function is to establish global processes. To optimize progress across a large and complex set of activities at the global level, standards, performance criteria, and processes for data sharing, communication, and convening must be established. The Enterprise will convene fora to address policy issues such IP, clinical trials, site development, and regulatory hurdles. And the third essential function is shared accountability. The partners in this alliance will need to create a culture of mutual accountability for the effective implementation of the scientific strategic plan. Since the Enterprise is not a single organization, a shared “way of doing business” is one of its most important defining traits. Articulating an explicit set of “working principles” is therefore crucial to the identity and smooth functioning of the Enterprise.

For the Enterprise as a whole the following conditions apply: (1) the central task is to develop and implement an ambitious scientific plan with the necessary scale, balance and sequence of activities, and structure to carry it out; (2) the plan must focus on critical roadblocks that would benefit substantially from global collaboration while fostering continued R&D by individuals, small groups, and individual networks; (3) the incentives holding the alliance together will include collaborative arrangements and structures that give people the resources, necessary critical mass, centralized facilities, common reagents, assays and technologies, and data they need to effectively remove critical roadblocks; (4) all activities will reflect the commitment to create an environment that maximizes the ability of participants to share data and biological materials, e.g., through the use of common standards for measurements and appropriate IP arrangements; and (5) the Enterprise also commits to working for rapid global access to a successful vaccine.

For participating investigators and organizations, key principles include (1) the willingness and desire to work in an open, collaborative fashion, sharing data and reagents in a collegial fashion, with the appropriate balance between productive competition and effective collaboration, and (2) the willingness and ability to devote the majority of their time to tackling these problems within a focused environment, completely committing to solve the problems at hand.

### Organizational structure of the Enterprise

**Figure 6:**
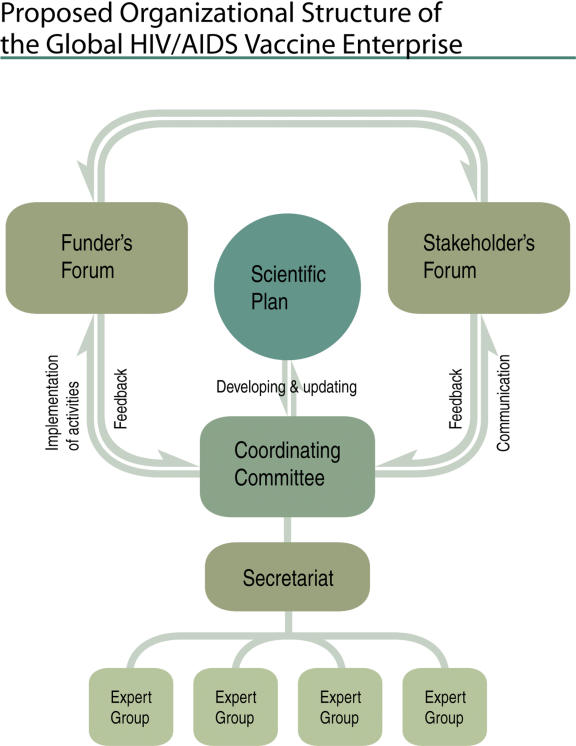
Proposed Organizational Structure of the Global HIV/AIDS Vaccine Enterprise (Illustration: Giovanni Maki)

The implementation of the scientific plan of the Enterprise will be overseen and supported by the organizational structure described in Figure 6.

The Coordinating Committee will facilitate all aspects of the Enterprise's activities. This committee consists of representatives of the Enterprise founders as well as additional scientific leaders selected from inside and outside the field of HIV vaccine research and development. The committee will develop procedures for term rotation and inclusion of new members, to ensure appropriate representation of all relevant partners, and will engage external stakeholders for advice, expertise, and assistance, appointing technical expert groups as needed. A Secretariat will provide logistical and administrative support to the Coordinating Committee and Enterprise partners. The BMGF will serve as Interim Secretariat until a permanent Secretariat is established.

The road to success will be a bumpy one.

The Funders Forum will be an open forum of sovereign, independent funding organizations, starting with a nucleus of those who already embrace the principles of the Enterprise and who are actively supporting or intend to support and fund HIV vaccine research and development. Members of the Funders Forum will be high-level decision makers within the ranks of funding organizations and governments, as close as possible to the source of resources. Since the Enterprise is not a discrete organization with a pool of money, funders will support specific areas using their own mechanisms, according to their own practices and policies, and following Enterprise principles. The scientific plan will provide guidance that may help funders better align existing resources but, more importantly, will facilitate the efficient and focused application of new resources as they become available. Multiple funders who wish to support a single Enterprise-defined project could form collaborative agreements, memoranda of understanding, or other forms of written agreement among themselves to outline their respective roles and responsibilities; address IP, program management, oversight, and other issues; and establish mechanisms for communication and conflict resolution. The funders with greatest flexibility could provide incentives for sharing reagents and data, and linking projects together, e.g., by supporting the additional work that nationally or regionally funded laboratories would need to undertake in order to participate in a global network, or by supporting a program to develop and share reagents.

In some cases, funders may wish to support an implementing organization that will take responsibility for managing the project and reporting back to the funder and other stakeholders. In other cases, funders may have the capability and capacity to play a substantial role in facilitating the project. In still other cases, funders may have the capability to assume a leadership role in overseeing the conduct of the activity, particularly in cases where the activity is well defined in advance.

In addition, an Annual Stakeholders Forum will be organized to bring together the broader community of scientists, policy makers, public health officials, and community representatives involved in the search for an HIV/AIDS vaccine. This meeting will serve as a forum to (1) update the broader community on Enterprise activities and progress, and (2) provide the community with a mechanism for feedback and dialog.

### Funding issues

Global expenditures on HIV vaccine research and development in 2002 were tentatively estimated to be on the order of US$624–670 million, the large majority (67.3%) provided by the public sector, followed by the philanthropic sector (17.4%) and industry (15.3%). An analysis of how those funds have been invested revealed that the large majority (43.1%) is being used in preclinical research activities, followed by clinical trials (28.2%), basic research (20.7%), cohort development and clinical trial infrastructure (6.5%), and vaccine education, advocacy, and policy development (1.4%) [27].

The largest funder of HIV vaccine research and development activities has been the NIH, with almost US$350 million in 2002. The NIH budget for HIV vaccine research has grown from less than US$50 million in 1996, to an estimated US$514.6 million for 2005, corresponding to 17.6% of the NIH total HIV-related research budget for 2005.

The Enterprise Coordinating Committee will analyze the additional financial requirements to fully implement the scientific plan of the Enterprise, and the Enterprise Secretariat will explore options to leverage these funds from the public and private sector. Initial estimates by Enterprise partners suggest that US$1.2 billion per year, or double the current expenditures on HIV vaccine research and development, will be needed. Although this amount may appear unrealistic at present, it would represent only a fraction of the total global expenditures in response to the AIDS pandemic and a very reasonable investment in view of the enormous social, political, and economic consequences of the pandemic. However, it is essential that the proposed increase in funding for HIV vaccine R&D be additional to existing AIDS expenditures, and not at the expense of current prevention, treatment, and care efforts.

The founding partners of the Enterprise, including the NIH, the BMGF, and the Wellcome Trust have already committed, or are considering committing, resources towards new initiatives that will begin to enact portions of the Enterprise scientific plan over the next six to nine months. Each funder will utilize their own funding processes and will align the design, scope, and scale of programs to those laid out in this plan. For example, the NIH National Institute of Allergy and Infectious Diseases will establish the Center for HIV Vaccine Immunology, which will target several scientific priorities identified here.

### Political support

As a sign of global recognition of the importance of better, more strategic coordination in the search for an HIV vaccine, the “Group of Eight” leading industrialized nations in June 2004 endorsed the goals of the Enterprise and agreed to review progress in implementation at its 2005 summit meeting in the United Kingdom [28]. Likewise, on October 19, 2004, Ministers of Health from seven European countries (France, Germany, Italy, the Netherlands, Spain, Sweden, and the United Kingdom) adopted a statement of intent to coordinate efforts to accelerate research for an HIV vaccine within the context of the global effort.

## Next Steps

With almost 5 million new HIV infections and 3 million AIDS deaths occurring every year worldwide, the development of a safe, effective, and accessible HIV vaccine represents one of the most urgent global public health needs. This global emergency led to the proposal to harness the power of science to find a definitive solution to one of the most catastrophic health problems of our time. The Global HIV/AIDS Vaccine Enterprise has evolved over the past 18 months from a concept proposed in a scientific journal by a cadre of researchers to a global consensus concerning the major scientific roadblocks facing HIV vaccine development, a strategic approach to address those roadblocks, and guiding principles for the plan's implementation in a manner and degree commensurate with the challenges at hand. Several organizations have already embraced the Enterprise concept and are moving to tackle portions of the scientific plan. Still, much more remains to be done. The road to success will be a bumpy one requiring the energy, commitment, and action of a wide number of government and non-governmental organizations globally. Recognizing the enormity of the roadblocks as well as the potential benefits of a safe and effective HIV vaccine, it is essential that many more organizations and agencies contribute additional expertise and resources and work together as a global community in a cooperative, collaborative, and transparent manner to fully implement the Enterprise scientific plan. 

## Abbreviations

BMGF: Bill & Melinda Gates Foundation
GLP: Good Laboratory Practices
IP: intellectual property
NIH: United States National Institutes of Health
R&D: research and development
SIV: simian immunodeficiency virus
UNAIDS: Joint United Nations Programme on HIV/AIDS
WHO: World Health Organization
